# Information and BMI limits for patients with obesity eligible for knee arthroplasty: the Swedish surgeons’ perspective from a nationwide cross-sectional study

**DOI:** 10.1186/s13018-022-03442-5

**Published:** 2022-12-19

**Authors:** Perna Ighani Arani, Per Wretenberg, Annette W-Dahl

**Affiliations:** 1 Department of Orthopedic Surgery, Örebro University Hospital, Örebro, Sweden; 2 Faculty of Medicine and Health, School of Medical Sciences, Örebro University, 702 81 Örebro, Sweden; 3 Department of Clinical Sciences Lund, Orthopedics, Faculty of Medicine, Lund University, 221 00 Lund, Sweden; 4 The Swedish Arthroplasty Register, Göteborg, Sweden

**Keywords:** Osteoarthritis, Knee arthroplasty, Obesity, Routines, BMI limit

## Abstract

**Background:**

In the past decades, the incidence of obesity has increased worldwide. This disease is often accompanied with several comorbidities and therefore, surgeons and anesthesiologists should be prepared to provide optimal management for these patients. The aim of this descriptive cross-sectional study was to map the criteria and routines that are used by Swedish knee arthroplasty surgeons today when considering patients with obesity for knee arthroplasty.

**Methods:**

A survey including 21 items was created and sent to all the Swedish centers performing knee arthroplasty. The survey included questions about the surgeons’ experience, hospital routines of preoperative information given and the surgeons’ individual assessment of patients with obesity that candidates for knee arthroplasty. Descriptive statistics were used to present the data.

**Results:**

A total of 203 (64%) knee surgeons responded to the questionnaire. Almost 90% of the surgeons claimed to inform their patients with obesity that obesity has been associated with an increased risk of complications after knee arthroplasty. Seventy-nine percent reported that they had an upper BMI limit to perform knee arthroplasty, a larger proportion of the private centers had a BMI limit compared to public centers. The majority of the centers had an upper BMI limit of 35.

**Conclusion:**

The majority of the knee arthroplasty surgeons in Sweden inform their patients with obesity regarding risks associated with knee arthroplasty. Most centers that perform knee arthroplasties in Sweden have an upper BMI limit.

## Introduction

Obesity has increased in the past few decades and is identified as a global epidemic [1, 2]. Patients with obesity often suffers from multiple comorbidities, such as cardiovascular diseases and diabetes mellitus [3, 4]. Therefore, surgeons and anesthesiologists should be prepared to provide optimal management of these patients.

Obesity is a prominent risk factor for osteoarthritis (OA) [5, 6] and has been shown to be a reason for the increasing incidence of total knee arthroplasty (TKA) [7, 8].

Despite the considerable benefits associated with knee arthroplasty, unfavorable outcomes do occur. Approximately 4% of patients with primary total knee arthroplasty due to OA experience an adverse event within 90 days [9], and 8% experience dissatisfaction after a knee arthroplasty [9, 10] in Sweden. These figures along with several other factors associated with complications, such as smoking and weight, must be taken into consideration when determining whether a patient is appropriate for surgery or not. However, the authors have not been able to find any previous studies on how the surgeons inform their patients with obesity about the potential risks with knee arthroplasty.

Therefore, the aim of this descriptive cross-sectional study was to map the criteria and routines used for patients with obesity when considering knee arthroplasty among Swedish knee arthroplasty surgeons. Furthermore, we evaluated how these patients are informed preoperatively about the potential risk of pre- and postoperative complications.

## Methods

A survey in Swedish, including 21 items, was developed and created by the authors. The survey was sent by mail to the 64 centers in Sweden performing knee arthroplasty in late April 2022 with a deadline to answer within one month. A reminder was sent by mail after approximately two weeks. The units performing knee arthroplasty in Sweden and a contact person of knee arthroplasty of each unit were obtained from Swedish Arthroplasty Register (SAR). Since the number of knee arthroplasty surgeons of each center was unknown, it was not possible to send the survey electronically.

The survey took approximately 2–3 min to complete. The survey included questions about the surgeons’ experience, routines of preoperative information of the hospital and the surgeons’ individual assessment of patients with obesity that candidates for knee arthroplasty (Appendix 1). A five-point Likert scale was used when appropriate, and some of the questions was answered with yes or no. The surgeons were also asked to state which hospital they worked at. An attachment that included more detailed information about the study and questions regarding the number of knee arthroplasty surgeons working on each center was enclosed in the letter to the contact person. The aim of this attachment was to collect the total number of knee arthroplasty surgeons on each center.

In order to collect the total number of non-responders, a phone call was made to all the centers which had not sent back the attachment of the survey by the end of the deadline, to collect the total number of knee arthroplasty surgeons on each center.

### Statistics

Descriptive statistics were used to present the data. Frequencies of the answers were presented in numbers and percentages, while Body Mass Index (BMI) limits were presented with median and interquartile range. Missing data were excluded from all analyses. Statistical Package for the Social Sciences was used for the statistical analyses.

## Results

### Response rates

A total of 319 knee arthroplasty surgeons were reported to work at the 64 knee arthroplasty centers in Sweden. However, several of the surgeons work temporarily at several units and in those cases were counted more than once. The number of knee surgeons is in line with a previous study that sent out questionnaires to Swedish knee arthroplasty surgeons [11]. In the current study, 48 out of 64 centers that perform knee arthroplasty responded. There was a higher response rate from the public centers (39/49) compared to the private centers (10/16). The number of responding knee surgeons was also higher at the public centers (72%) compared to private centers (38%). A total of 203 (64%) knee surgeons responded to the questionnaire. The majority of the respondents had been specialists for more than 8 years (69%) and spent more than 8 years working with knee arthroplasty as a specialist (60%) (Table 1). One hundred and fifty-three out of 203 of the responders mentioned that knee revisions were performed.

**Table 1:** Characteristics of responders

Question	Alternative	Answers
How many years have you been an orthopedic surgeon?	0–3 years, *n* (%)	21 (10)
	3–8 years, *n* (%)	43 (21)
	> 8 years, *n* (%)	139 (69)
How many years have you worked with knee arthroplasty surgery as an orthopedic specialist?	0–3 years, *n* (%)	34 (17)
	3–8 years, *n* (%)	47 (23)
	> 8 years, *n* (%)	121 (60)
How many primary knee arthroplasty surgeries do you perform each year?	0–10, *n* (%)	12 (6)
	11–25, *n* (%)	33 (16)
	26–50, *n* (%)	59 (29)
	> 50, *n* (%)	98 (49)
Is revisions of knee arthroplasty performed at your clinic?	No, *n* (%)	50 (25)
	Yes, *n* (%)	153 (75)
Do you perform revisions of knee arthroplasty?	No, *n* (%)	103 (51)
	Yes, *n* (%)	99 (49)
How many percent of your total orthopedic work is comprised of knee arthroplasty patients?	About 25%, *n* (%)	96 (47)
	About 50%, *n* (%)	75 (37)
	About 75%, *n* (%)	27 (13)
	About 100%, *n* (%)	5 (3)

### Information and routines

**Fig. 1:**
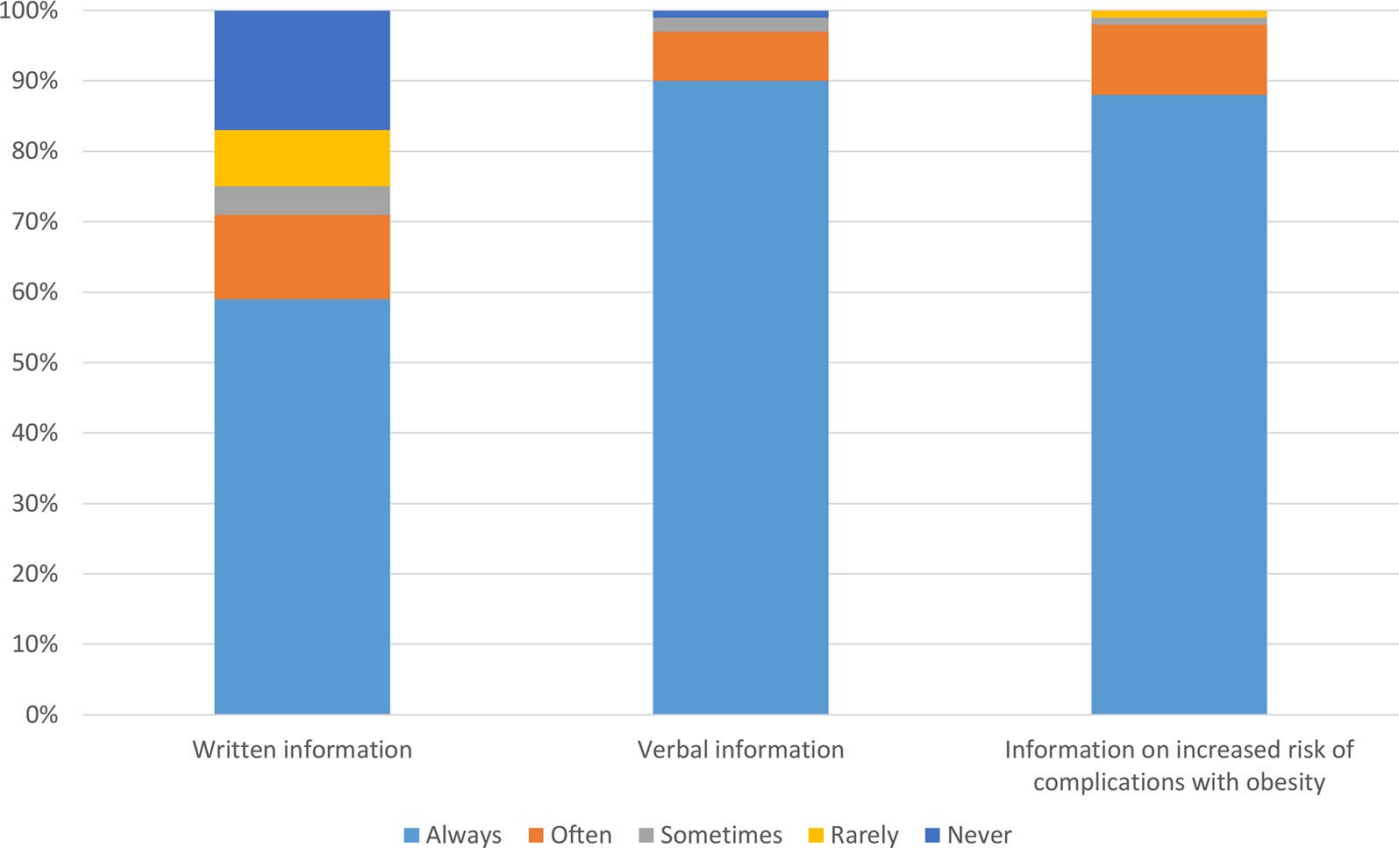
General routines

**Fig. 2:**
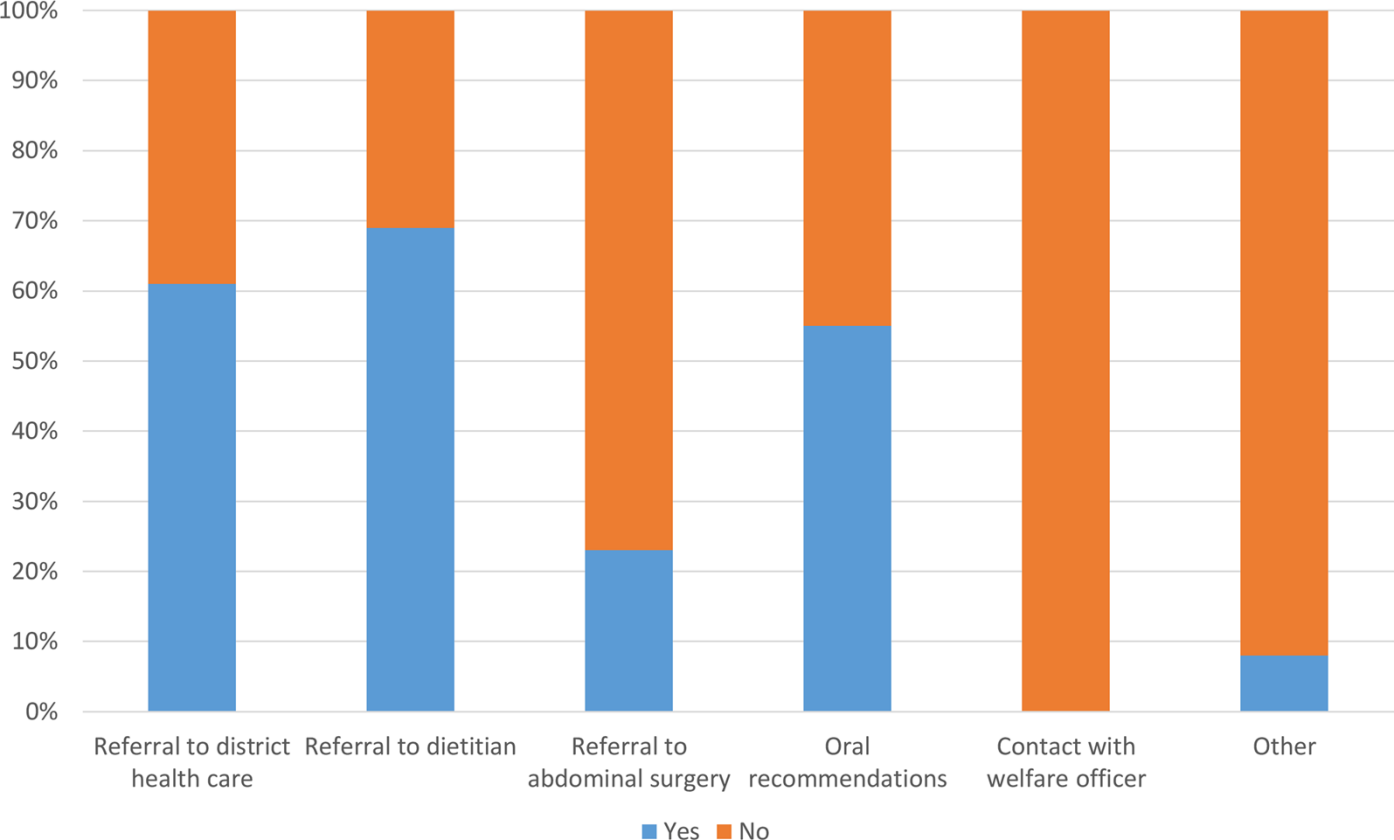
Specific routines if weight loss is recommended

All the responding knee arthroplasty surgeons reported that they inform the patients about surgical risks and complications after knee arthroplasty. Almost 90% of the surgeons inform their patients with obesity that obesity has been associated with an increased risk of complications after knee arthroplasty (Table 2, Fig. 1). Seventy-nine percent of all the responders reported that they had an upper BMI limit to perform knee arthroplasty (Table 3). There were a larger proportion of the responders at the private centers who stated that they had an upper BMI limit compared to the responders at the public centers (87% and 73% respectively). The majority of surgeons answered that their center had 35 as an upper BMI limit in both the private and public centers (59% and 62% respectively). However, in some centers the surgeons answered differently concerning the upper BMI limit. Seventy-five percent of all the responders reported that their center had a routine to recommend weight loss for patients with obesity that are eligible for knee arthroplasty (Table 3, Fig. 2). Approximately 76% of the responders reported that they rarely or never required additional blood tests if a patient with obesity is scheduled for knee arthroplasty (Table 3).

**Table 2:** General routines of knee arthroplasty centers of Sweden

Question	Alternative	Answers
Who is informing the patients about risks and/or complications regarding knee arthroplasty? You as a knee surgeon	No, *n* (%)	0 (0)
	Yes, *n* (%)	203 (100)
Who is informing the patients about risks and/or complications regarding knee arthroplasty? Nurse	No, *n* (%)	177 (87)
	Yes, *n* (%)	26 (13)
Who is informing the patients about risks and/or complications regarding knee arthroplasty? Physiotherapist	No, *n* (%)	190 (94)
	Yes, *n* (%)	13 (6)
Who is informing the patients about risks and/or complications regarding knee arthroplasty? Other	No, *n* (%)	201 (99)
	Yes, *n* (%)	2 (1)
Is your clinic using written information where risks and/or potential complications of knee arthroplasty is described?	Always, *n* (%)	118 (59)
	Often, *n* (%)	25 (12)
	Sometimes, *n* (%)	7 (4)
	Rarely, *n* (%)	16 (8)
	Never, *n* (%)	35 (17)
Do you use verbal information to describe risks, complications and benefits of knee arthroplasty?	Always, *n* (%)	183 (90)
	Often,* n* (%)	15 (7)
	Sometimes, *n* (%)	4 (2)
	Rarely, *n* (%)	0 (0)
	Never, *n* (%)	1 (1)
Do you inform that obesity could be an increased risk for complications of knee arthroplasty to patients with obesity?	Always, *n* (%)	178 (88)
	Often, *n* (%)	21 (10)
	Sometimes, *n* (%)	3 (1)
	Rarely, *n* (%)	1 (1)
	Never, *n* (%)	0 (0)

**Table 3:** Specific routines concerning obesity of knee arthroplasty centers of Sweden

Question	Alternative	Answers
Does your clinic have an upper limit of BMI for knee arthroplasty?	No, *n* (%)	51 (25)
	Yes, *n* (%)	152 (75)
Private clinics	No, *n* (%)	4 (13)
	Yes, *n* (%)	26 (87)
Public clinics	No, *n* (%)	47 (27)
	Yes, *n* (%)	126 (73)
If yes, what is the limit?	BMI limit, Median [IQR]	35 [35,40]
Private clinics	BMI limit, Median [IQR]	35 [35,40]
Public clinics	BMI limit, Median [IQR]	35 [35,40]
If yes on item 12, do you adhere to the BMI limit?	Always, *n* (%)	71 (46)
	Often, *n* (%)	75 (49)
	Sometimes, *n* (%)	7 (4)
	Rarely, *n* (%)	1 (1)
	Never, *n* (%)	0 (0)
How often do you schedule a patients with BMI ≥ 35 for knee arthroplasty?	Always, *n* (%)	4 (2)
	Often, *n* (%)	19 (10)
	Sometimes, *n* (%)	65 (32)
	Rarely, *n* (%)	89 (44)
	Never, *n* (%)	25 (12)
If you deny a patient for knee arthroplasty due to obesity, what is the reason?	Surgery becomes more difficult technically, *n* (%)	0 (0)
	Too many risks, *n* (%)	118 (58)
	Both above, *n* (%)	67 (33)
	Other, *n* (%)	2 (1)
	Do not deny due to obesity, *n* (%)	15 (8)
If you deny a patient a knee arthroplasty due to obesity, on what does your decision rely on? Previous studies	No, *n* (%)	34 (17)
	Yes, *n* (%)	168 (83)
If you deny a patient for knee arthroplasty due to obesity, on what does your decision rely on? The clinics experiences	No, *n* (%)	104 (51)
	Yes, *n* (%)	98 (49)
If you deny a patient for knee arthroplasty due to obesity, on what does your decision rely on? Own experiences	No, *n* (%)	126 (62)
	Yes, *n* (%)	76 (38)
If you refuse a patient for knee arthroplasty due to obesity, on what does your decision rely on? Do not deny due to obesity	No, *n* (%)	185 (92)
	Yes, *n* (%)	17 (8)
Does your clinic have a routine to recommend weight loss for patients with obesity that are eligible for knee arthroplasty?	No, *n* (%)	51 (25)
	Yes, *n* (%)	151 (75)
If yes, what routines do you have on your clinic? Referral to district health care	No, *n* (%)	63 (39)
	Yes, *n* (%)	100 (61)
If yes, what routines do you have on your clinic? Referral to dietitian	No, *n* (%)	113 (69)
	Yes, *n* (%)	50 (31)
If yes, what routines do you have on your clinic? Referral to abdominal surgery for possible bariatric surgery	No, *n* (%)	125 (77)
	Yes, *n* (%)	38 (23)
If yes, what routines do you have on your clinic? Oral recommendation	No, *n* (%)	74 (45)
	Yes, *n* (%)	89 (55)
If yes, what routines do you have on your clinic? Contact to welfare officer	No, *n* (%)	163 (100)
	Yes, *n* (%)	0 (0)
If yes, what routines do you have on your clinic? Other	No, *n* (%)	150 (92)
	Yes, *n* (%)	13 (8)
Do you refer patients for possible bariatric surgery for patients with BMI > 35 before a knee arthroplasty?	Always, *n* (%)	7 (3)
	Often, *n* (%)	18 (9)
	Sometimes, *n* (%)	50 (25)
	Rarely, *n* (%)	44 (22)
	Never, *n* (%)	82 (41)
If a patient with obesity is scheduled for knee arthroplasty, do you require additional blood tests in excess of the regular blood tests?	Always, *n* (%)	9 (5)
	Often, *n* (%)	11 (5)
	Sometimes, *n* (%)	27 (13)
	Rarely, *n* (%)	72 (36)
	Never, *n* (%)	84 (41)

We found similar answers in providing information preoperatively regarding obesity (always, often, sometimes, rarely or never) depending on the surgeon’s experience as an orthopedic specialist (0–3 years, 3–8 years, > 8 years). Furthermore, we found similar answers in adherence to the BMI limits when scheduling patients with BMI ≥35 for knee arthroplasty depending on the surgeon’s experience as an orthopedic specialist.

## Discussion

This descriptive study indicates that all the responding knee arthroplasty surgeons provide their patients with general information preoperatively regarding the risks with knee arthroplasty, of whom almost 90% inform their patients about the increased risks with obesity associated with knee arthroplasty. The majority of the clinics also had an upper limit of BMI for knee arthroplasty.

Obesity has been linked to worse outcomes after TKA and patients with obesity have been shown to have an increased risk of revision after TKA in several studies [12–17]. Sezgin et al. reported that obesity is associated with an increased risk of revision for all causes and for infection, but not for revision for other causes than infection [18]. In a study investigating the association between metabolic syndrome and pre- and postoperative complications in total joint arthroplasty (TJA), showed a statistically significant increased risk in complications in patients with metabolic syndrome compared to those without metabolic syndrome. Elevated BMI was the element of metabolic syndrome that had the largest impact on postoperative complication rate. In addition, BMI ≥ 30 was found to have a statistically significant increase in surgical time and length of stay compared to patients with normal weight [19]. Obesity is also associated with an increased risk of developing OA that may require surgery at younger age [20]. Furthermore, patients with obesity require comprehensive preoperative evaluation due to the increased risks perioperativly [21]. Roche et al. demonstrated that the cost of treating patients with obesity with TKA rises as BMI deviates from normal, as well as the incidence of revisions [15]. The presence of an upper BMI limit appeared to be present more often at the private hospitals compared to public hospitals. In addition, a lower proportion of patients with BMI above 35 is operated on at the private centers compared to public centers [9]. One possible explanation to this may be that obesity is often accompanied with other comorbidities and can therefore increase the need of more advanced postoperative care. One of the responding surgeons mentioned that their limit was due to the restricted resources of intensive care unit at their private hospital.

A study published by Ledford et al. [22] reported that the percentage of body fat may be a more adequate method than BMI in determining risks related to TJA performed in patients with obesity. However, measuring percentage of body fat is a more resource-demanding and time-consuming procedure compared to calculating BMI.

Weight loss is one of the first-line treatments of knee OA according to the Swedish national guidelines [23], and should be routinely recommended to patients with obesity before considering surgery. Weight loss have been shown to improve the symptoms of knee complaints [24]. Approximately 75% of the knee arthroplasty surgeons reported that they have routines to recommend weight loss to patients with obesity when considering knee arthroplasty. Bariatric surgery (BS) has been shown to be the most effective method in achieving significant long-term weight loss for patients with obesity in comparison with nonsurgical interventions [25]. A recent randomized control trial, analyzing the weight change after TKA in patients with prior BS or not, demonstrated that 12 of the 41 patients who had received BS did no longer require a TKA since their symptoms had improved [26]. The question arises if we should routinely recommend patients with obesity that fail to lose weight through other methods, the possibility to perform BS prior to their knee arthroplasty. In our study, we found that 23% of the knee arthroplasty surgeons refer the patients with obesity to an abdominal surgeon for the consideration of a possible BS. However, a systematic review published in 2016 showed that BS prior to TJA did not reduce the adverse events or improve the clinical outcome for most perioperative outcomes [27]. Furthermore, other studies have not shown any improvement in patients with BS prior to their TKA regarding the risk of revision as an outcome [28, 29]. The fact that patients undergoing gastric bypass may remain in a catabolic state two years after the surgery, could be an explanation [30]. However, it is difficult to draw conclusions between the association of BS and outcomes postoperatively to TKA since there is a lack of studies that take the numerous accompanied comorbidities into consideration.

A cross-sectional study assessing preoperative nutritional status in patients who were candidates to BS found that malnutrition, particularly in vitamin D and iron, was pervasive in these patients [31]. Mosli et al. found that obesity is associated with higher odds of hypoalbuminemia [32]. Another study showed that hypoalbuminemia and obesity grade II together are reliable predictors of periprosthetic joint infection after elective TKA, whereas overweight and obesity grade I were not associated with such complications [33]. Nelson et al. found that hypoalbuminemia was more associated with major complications after TKA than morbid obesity [34]. In the current study, the majority of the responders reported that they rarely or never prescribed additional blood samples to patients with obesity. However, the questionnaire did not take in to consideration that the clinics may already obtain albumin sample as a routine from all the patients.

A previous Swedish study analyzed patients operated with knee arthroplasty between 1981 and 1997 and found that 8% of the patients were not satisfied regarding their knee surgery 2–17 years postoperatively. The satisfaction was affected by the preoperative diagnosis leading to surgery, e.g. rheumatic disease and OA. However, preoperative BMI was not taken into consideration [10]. Another Swedish study found that the majority of both patients with or without prior BS to TKA were satisfied or very satisfied after their TKA and found no difference in one-year postoperative pain or ADL function to TKA between the two cohorts. However, the analysis was adjusted for BMI before TKA in the patients with prior BS to TKA [35]. Furthermore, a study from 2019 did not find any difference in improvement in PROs one year after TKA depending on the BMI [36].

When extracting the knee surgeons who answered that they refuse a patient for knee arthroplasty due to obesity, 83% reported that they based their decision on previous studies. There are limited prospective interventional studies available regarding obesity preoperatively to knee arthroplasty. The evidence is not clear regarding the use of an upper BMI limit as an inclusion criterion to perform knee arthroplasty in order to improve postoperative outcomes after TKA. The majority of surgeons in both the private and public centers answered that their center had 35 as an upper BMI limit. However, all private centers except one reported knee arthroplasties in patients with BMI ≥ 35, and only two of the public centers reported no knee arthroplasties in patients with BMI ≥ 35 in 2021. According to the SAR, the prevalence of performing knee arthroplasty in patients with morbid obesity (BMI ≥ 40) is low (1%) [9].

This descriptive cross-sectional study based on a questionnaire has limitations of this type of design. In addition, the questionnaire was created by the authors which suffers from the absence of validation and psychometric analysis of the survey. However, to the authors knowledge, there are no validated questionnaires that evaluate these type of questions. Furthermore, this is a hypothesis generating study with the aim to yield ideas for further studies. The responses obtained in this current study could be considered to be representative of the knee arthroplasty surgeons in Sweden due to a relatively high response rate. When the answers were compiled, it appeared from the comments at the end of the survey that several surgeons did not know the correct definition of revision and the difference between overweight and obesity, which could have been more clearly explained in the survey. This effects the answers in questions 5 and 6 (Appendix 1). Finally, this study evaluated the frequency of surgeons who provided information to their patients and excludes the quality of the information given. The exact information that is given by the surgeons or received by the patients could be of value as it possibly affects both the patients’ expectations, and also their satisfaction rates. Considering that this is a descriptive cross-sectional study with the aim to map the criteria and routines using a questioner that has not been validated, the authors want to be careful to give any recommendations using only this paper. Further, studies with a higher grade of evidence are probably required before any type of recommendations are given regarding the management of this patient population.

## Conclusion

There is limited evidence on how to manage patients with obesity preoperatively to knee arthroplasty, and whether to have an upper BMI limit or not. This descriptive study indicates that the majority of the knee arthroplasty surgeons in Sweden inform their patients with obesity regarding risks of knee arthroplasty. Furthermore, most centers that perform knee arthroplasties in Sweden have an upper BMI limit. Further studies on this topic are encouraged by the authors.

## Data Availability

The data are available at reasonable request.

## Appendix 1

**Table:**

Question in original language	Translated question	Alternative (original/translated)
Vilken klinik arbetar Du på?	What clinic do you work at?	Fri text/Free text
Hur många år har Du varit ortopedspecialist?	How many years have you been an orthopedic surgeon?	0–3 år/years
		3–8 år/years
		> 8 år/years
Hur många år har Du arbetat med knäproteskirurgi som specialist?	How many years have you worked with knee arthroplasty surgery as an orthopedic specialist?	0–3 år/years
		3–8 år/years
		> 8 år/years
Hur många primära knäprotesoperationer gör Du per år?	How many primary knee arthroplasty surgeries do you perform each year?	0–10
		11–25
		26–50
		> 50
Utförs knäprotesrevisioner på kliniken Du arbetar på?	Is revisions of knee arthroplasty performed at your clinic?	Nej/No
		Ja/Yes
Genomför Du knäprotesrevisioner?	Do you perform revisions of knee arthroplasty?	Nej/No
		Ja/Yes
Hur många procent av Ditt totala ortopedarbete omfattas av knäprotes-patienter?	How many percent of your total orthopedic work is comprised of knee arthroplasty patients?	Ca/About 25%
		Ca/About 50%
		Ca/About 75%
		Ca/About 100%
Vem informerar patienterna om risker och/eller potentiella komplikationer med knäproteskirurgi?	Who is informing the patients about risks and/or complications regarding knee arthroplasty?	Du som knäproteskirurg/ You as a knee surgeon
		Sjuksköterska/Nurse
		Fysioterapeut/physiotherapist
		Annan/Other
Använder Din klinik skriftlig information där risker och/eller potentiella komplikationer med knäproteskirurgi beskrivs?	Is your clinic using written information where risks and/or potential complications of knee arthroplasty is described?	Alltid/Always
		Ofta/Often
		Ibland/Sometimes
		Sällan/Rarely
		Aldrig/Never
Använder Du muntlig information för att beskriva risker, komplikationer och vinster med knäproteskirurgi?	Do you use verbal information to describe risks, complications and benefits of knee arthroplasty?	Alltid/Always
		Ofta/Often
		Ibland/Sometimes
		Sällan/Rarely
		Aldrig/Never
Informerar Du om att fetma kan innebära ökad risk för komplikationer vid knäproteskirurgi till patienter med obesitas?	Do you inform that obesity could be an increased risk for complications of knee arthroplasty to patients with obesity?	Alltid/Always
		Ofta/Often
		Ibland/Sometimes
		Sällan/Rarely
		Aldrig/Never
Har Din klinik en övre gräns på BMI för knäproteskirurgi?	Does your clinic have an upper limit of BMI for knee arthroplasty?	Nej/No
		Ja/Yes
Om Ja, vilken är den övre gränsen?	If yes, what is the limit?	Fri text/Free text
Om ja på fråga 12, följer Du BMI-gränsen?	If yes on item 12, do you adhere to the BMI limit?	Alltid/Always
		Ofta/Often
		Ibland/Sometimes
		Sällan/Rarely
		Aldrig/Never
Hur ofta sätter Du upp patienter med BMI ≥ 35 för knäproteskirurgi?	How often do you schedule a patients with BMI ≥ 35 for knee arthroplasty?	Alltid/Always
		Ofta/Often
		Ibland/Sometimes
		Sällan/Rarely
		Aldrig/Never
Om Du nekar en patient knäproteskirurgi på grund av obesitas, vad är anledningen?	If you deny a patient for knee arthroplasty due to obesity, what is the reason?	Försvårar operationen tekniskt/Surgery becomes more difficult technically
		För många risker/Too many risks
		Båda ovanstående/Both above
		Annat/Other
		Nekar inte på grund av obesitas/Do not deny due to obesity
Om Du nekar en patient knäproteskirurgi på grund av obesitas. Vad grundar du ditt beslut på? Flera alternativ kan ringas in	If you deny a patient a knee arthroplasty due to obesity, on what does your decision rely on?	Vetenskaplig dokumentation/Previous studies
		Klinikens erfarenheter/The clinics experiences
		Egen erfarenhet/Own experiences
		Nekar inte pga obesitas/Do not deny due to obesity
Har Din klinik rutin på att rekommendera viktminskning för patienter med obesitas som är aktuella för knäproteskirurgi?	Does your clinic have a routine to recommend weight loss for patients with obesity that are eligible for knee arthroplasty?	Nej/No
		Ja/Yes
Om Ja, vilka rutiner har Ni på kliniken?	If yes, what routines do you have on your clinic?	Remiss till vårdcentralen/
		Remiss till dietist/
		Remiss till kirurgen för ev. viktminskningskirurgi/
		Muntlig rekommendation/
		Kurator kontakt/
		Annat/
Skickar Du remiss för bedömning av eventuell fetmakirurgi för patienter med BMI > 35 före en knäprotesoperation?	Do you refer patients for possible bariatric surgery for patients with BMI > 35 before a knee arthroplasty?	Alltid/Always
		Ofta/Often
		Ibland/Sometimes
		Sällan/Rarely
		Aldrig/Never
Om en patient med obesitas sätts upp för en knäprotesoperation, ordinerar Du andra prover/undersökningar utöver de rutinmässiga?	If a patient with obesity is scheduled for knee arthroplasty, do you require additional blood tests in excess of the regular blood tests?	Alltid/Always
		Ofta/Often
		Ibland/Sometimes
		Sällan/Rarely
		Aldrig/Never
Något annat Du önskar framföra?	Something else you want to comment?	Fri text/Free text

## Abbreviations

BMI: Body mass index
BS: Bariatric surgery
SAR: Swedish Arthroplasty Register
TJA: Total joint arthroplasty
TKA: Total knee arthroplasty
